# Effects of antimicrobial peptide and tributyrin on fecal microflora and blood indices of female calves

**DOI:** 10.1002/fsn3.3483

**Published:** 2023-06-12

**Authors:** Junling Gao, Jianan Dong, Zhe Sun, Tao Wang, Yanling Guan, Yue Sun, Guixin Qin, Xuefeng Zhang, Yuguo Zhen

**Affiliations:** ^1^ College of Animal Science and Technology, JLAU‐Borui Dairy Science and Technology R&D Center, Key Laboratory of Animal Nutrition and Feed Science of Jilin Province, Key Laboratory of Animal Production Product Quality and Security Ministry of Education Jilin Agricultural University Changchun China; ^2^ Postdoctoral Scientific Research Workstation, Feed Engineering Technology Research Center of Jilin Province Changchun Borui Science & Technology Co., Ltd Changchun China; ^3^ College of Life Science, Jilin Agricultural University Changchun China; ^4^ Institute of Animal Husbandry and Special Animal Science Heilongjiang Academy of Land Reclamation Sciences Harbin China

**Keywords:** antimicrobial peptide, blood indices, calves, fecal microflora, tributyrin

## Abstract

This study evaluated the effects of antimicrobial peptide (AMP) and tributyrin (TB) on dairy calves in terms of growth performance, immunity, oxidative stress, and intestinal microflora. A total of 40 female calves were divided into four treatment groups (*n* = 10): basal diet +0.015% essential oil, basal diet +0.03% AMP, basal diet +0.15% TB, and basal diet +0.03% AMP + 0.15% TB. AMP and TB supplementation increased the average daily gain (ADG) and weaning weight, while reducing diarrhea occurrence. Additionally, AMP and TB supplementation reduced the levels of reactive oxygen species (ROS) and malonaldehyde (MDA), while increasing superoxide dismutase (SOD) levels and serum immunoglobulin M (IgM) levels. However, the combined use of AMP and TB did not significantly affect the average daily feed intake, ADG, weaning weight, or diarrhea incidence but decreased ROS levels, while increasing SOD levels as well as MDA and IgM levels. Moreover, AMP and TG supplementation increased the relative abundance of several beneficial fiber‐ and mucin‐degrading bacteria in the gut, in contrast to combined AMP and TB supplementation. The 16S rRNA results showed that AMP supplementation significantly increased the relative abundance of *Rikenellaceae_RC9_gut_group*, *Ruminococcaceae_UCG‐014* and *[Eubacterium]_coprostanoligenes group* (*p* < .01), and significantly decreased the relative abundance of *Ruminococcaceae_UCG‐005* and *Christensenellaceae_R‐7_group* (*p* < .01). The TB supplementation significantly increased the abundances of *Rikenellaceae_RC9_gut_group* and *Ruminococcaceae_UCG‐005* (*p* < .01), and significantly decreased the relative abundances of *Ruminococcaceae_UCG‐014*, *[Eubacterium]_coprostanoligenes group* and *Christensenellaceae_R‐7_group* (*p* < .01). The combined use of AMP and TB significantly increased the relative abundance of *Rikenellaceae_RC9_gut_group* and *Bacteroides* (*p* < .01), and significantly decreased the relative abundance of *Ruminococcaceae_UCG‐014*, *[Eubacterium]_coprostanoligenes group* and *Christensenellaceae_R‐7_group* (*p* < .01). In summary, diets supplemented with either AMP or TB improved the intestinal microflora, growth performance, and health of weaned calves, but combined use was detrimental to calf performance.

## Graphical abstract

This study explores the benefits of dietary supplementation with antimicrobial peptides (AMPs) and tributyrin on the growth performance, immunity, oxidative stress, and intestinal microflora of dairy calves. The results indicate that diets supplemented with either AMPs or tributyrin can improve the intestinal microflora, growth performance, and health of weaned calves, but combined use of AMPs with tributyrin is detrimental to calf performance.

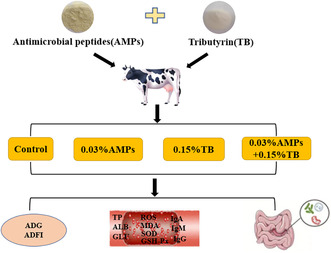

## 1 INTRODUCTION

Calves' cultivation is the key to the overall development of future cattle herds, but calves are highly susceptible to disease and even death during pre‐weaning. Urie et al. (2018) found that about 30% of deaths are caused by calves' intestinal diseases. In addition, calves are susceptible to other diseases during weaning stress, posing a threat to their health. Therefore, antibiotics will be fed to calves to enhance their immune system and promote healthy intestinal development. But the abuse of antibiotics has led to many undesirable consequences in recent years (Dibner & Richards, 2005; Mgr & Jcahb, 2017). To address this issue, the European Union implemented a “feed ban” policy in 2006, prohibiting the use of antibiotics as animal feed additives to promote animal growth (Brown et al., 2017). Therefore, current research is focused on developing green, safe, and effective alternatives to antibiotics in animal feed. Although increasing evidence supports the use of plant essential oils as growth promoters (Franz et al., 2010), extracting their active ingredients can be challenging and their mechanisms of action remain unclear.

Antimicrobial peptide (AMP), also known as “host defense peptide,” displays broad‐spectrum antifungal (Rautenbach et al., 2016), antibacterial (Rima et al., 2021), antiviral (Jenssen et al., 2006), and antiparasitic properties (Lacerda et al., 2016) and is less susceptible to bacterial resistance (Zhang et al., 2022). In addition, studies have shown that supplementation with AMP improves animal production performance (Bao et al., 2009), adjusts the balance of the gastrointestinal microflora (Xiao et al., 2015), and improves immune (Guo et al., 2021) and antioxidant functions (Tang et al., 2009). Therefore, AMP has become an antibiotic alternative with great potential. Tributyrin (TB) is a short‐chain fatty acid ester that can be broken down into butyric acid and glycerol by pancreatic lipases in the small intestine (Miyoshi et al., 2011; Vinolo et al., 2012). In addition, TB has no off‐flavor and can bypass the stomach to reach the hind gut (Augustin et al., 2011). This key property ensures that TB plays an important role in regulating gut homeostasis. Apart from its beneficial effects on the intestinal tract, TB supplementation can reportedly promote animal growth (Wang et al., 2019), improve the immune response (Jang et al., 2017), and regulate the structure and composition of the gut microbiota (Piva et al., 2002; Yang et al., 2018). One study indicates that TB may have a positive impact on rumen microbial protein production and fermentation (Ren et al., 2018). Notably, the gut microbiota also affects the digestion and the absorption of nutrients.

To date, most studies investigating supplementation with AMP and TB have focused on monogastric animals, whereas relatively few studies have been conducted on ruminants. Therefore, this study aimed to explore the effects of dietary AMP and TB on the growth performance, blood indicators, and fecal microflora of calves, thus providing a theoretical basis for the application of AMP and TB as feed additives to improve calf breeding.

## 2 MATERIALS AND METHODS

### 2.1 Experimental design

The experimental procedures were performed in accordance with the *Guidelines for the Care and Use of Experimental Animals* of Jilin Agricultural University (JLAU‐ACUC2017‐006). The feeding experiment was conducted at a commercial dairy farm from September 2020 to May 2021. A total of 40 female calves (average initial birth weight 40.5 + 5.5 kg) were randomly allocated to four treatment groups (*n* = 10). The control group (CON) was fed a basal diet supplemented with 0.015% essential oil (Phode), the AMP group was fed a basal diet supplemented with 0.03% AMP, the TB group was fed a basal diet supplemented with 0.15% TB, and the AMP&TB group was fed a basal diet supplemented with 0.03% AMP and 0.15% TB. AMP was provided by Yinghuier Biotechnology Co., Ltd. According to the product directions, AMP consists of 19 amino acid residues (sequence: Gly‐Gly‐Val‐Gly‐Lys‐Ile‐Ile‐Glu‐Tyr‐Phe‐Ile‐Gly‐Gly‐Gly‐Val‐Gly‐Arg‐Tyr‐Gly) and is in the shape of a circular folded lasso with a very stable nature of the peptide (including: alkali resistance, acid resistance, enzymatic hydrolysis resistance, high temperature resistance). And it was known for immune stimulation. TB was provided by Perstorp. Calves were separated from their dams immediately after birth and artificially fed 2–3 L colostrum within 1 h, followed by a second colostrum feeding after 6 h. The average Brix value of colostrum was 26.2 during this experiment. Calves were only given colostrum with Brix values more than 22. The calves were transferred to individual pens on the second day, had free access to water, and were fed pasteurized regular milk until day 65, as shown in Table 1.

**TABLE 1:** Quantity of milk fed to calves between 4 and 65 days.

Age	Number of feedings per day	Quantity of milk fed (mL/time)
Day 4–7	3	1750
Day 8–10	3	2000
Day 11–15	3	2250
Day 16–20	3	2500
Day 21–25	3	3000
Day 26–30	3	3250
Day 31–35	3	4000
Day 36–40	3	4000
Day 41–45	3	4000
Day 46–50	3	3250
Day 51–55	3	3000
Day 56–57	3	2500
Day 58–59	2	3000
Day 60–61	2	2000
Day 62–63	1	1000
Day 64–65	1	1000

To one set of pellets, AMP were added. To another set of pellets, TB was added. To a final set, both AMP and TB were added. Three groups of calves (all except the CON group) were fed this diet, one type of diet per group, from days 4 to day 65. Subsequently, the weaned calves were transferred to pens, 10 calves per pen, and fed a diet without the above supplementation from day 65 to day 180. The ingredient composition and nutritional values of the diets are presented in Table 2.

**TABLE 2:** Composition and nutrition levels of diets for days 4–180.

Ingredients	Contents (%)	Chemical composition	Contents (%)
Days 4–65			
Corn	29.70	Dry matter (%)	89.49
Bean pulp	28.00	Crude protein (%)	21.01
DDGS	6.00	Ash (%)	6.10
Corn bran	21.10	Ca (%)	0.90
Wheat middlings	3.00	P (%)	0.55
Molasses	2.00	ME/(MJ kg^−1^)	16.05
Extruded soybeans	5.00		
Premix^a^	5.20		
Days 65–180			
Corn	33.00	Starch (%)	29.95
Bean pulp	13.00	Crude protein (%)	18.37
Cottonseed meal 46%	7.30	Ash (%)	7.08
Corn germ meal (sol.)	4.00	Ca (%)	0.90
Wheat bran	7.00	NDF/ADF (%)	2.91
Domestic low‐fat DDGS	11.00	ME/(MJ kg^−1^)	12.68
Corn bran	15.20		
Wheat middlings	3.00		
Premix^a^	6.50		

Abbreviations: ADF, acid detergent fiber; Ca, calcium; DDGS, dried distillers' grains with solubles; ME, metabolizable energy; NDF, neutral detergent fiber.

^a^ Premix contained yeast culture, calcium hydrogen phosphate, calcium carbonate, sodium chloride, five grains peptide, milk flavoring agent, vitamins, trace elements, etc.

### 2.2 Sample collection and measurements

#### 2.2.1 Growth performance and diarrhea incidence

The calves were weighed on days 0, 30, 65, 90, 120, and 180 and the daily feed intake of each calf was recorded to calculate the average daily gain (ADG) and average daily feed intake (ADFI).

Fecal appearance was assessed on a daily basis using the following scale, as previously described (O'Shea et al., 2014): 1 = watery feces; 2 = loose feces and manure pile below 2.5 cm; 3 = the height of the manure pile is 3–4 cm, with a shallow pit in the middle; 4 = Thick feces and height of manure pile over 4 cm; 5 = hard feces and height of the manure pile is 5–10 cm. The average daily fecal score was calculated for each calf. Calves with fecal scores of ≤2 were considered to be diarrhetic.

Diarrhea incidence:

$$d=\frac{a}{b\times c}\times 100\%$$

where a is the number of calves with diarrhea, b is the total number of calves, c is the number of experiment days, and d is the incidence of diarrhea.

#### 2.2.2 Immunity and antioxidant indices in serum

Blood samples were collected from each calf using a 5‐mL vacutainer on days 45, 60, and 75. Each blood sample was centrifuged at 2264 *g* for 10 min to collect the serum, which was stored at −20°C until analysis. Serum immunoglobulins A (IgA), immunoglobulins M (IgM), and immunoglobulins G (IgG), as well as serum antioxidant indices glutathione peroxidase (GSH‐Px), superoxide dismutase (SOD), malondialdehyde (MDA), and reactive oxygen species (ROS), were measured using enzyme‐linked immunosorbent assay (ELISA) kits (Shanghai Enzyme‐linked Biotechnology, Co., Ltd.).

#### 2.2.3 Intestinal microflora

Fecal samples were collected by rectal stimulation from four randomly selected calves in each group on day 60. The fecal samples were stored in sterile tubes in liquid nitrogen at −80°C until analysis. The intestinal microflora of the fecal samples was analyzed by Parsono Biotechnology Co., Ltd.

The V3‐V4 region of the 16S rRNA gene was amplified with the forward primers 338F (5'‐ACTCCTACGGGAGGCAGCA‐3′) and reverse primers 806R (5'‐GGACTACHV‐GGGTWTCTAAT‐3′). On an Illumina MiSeq platform, the acquired DNA fragments underwent paired‐end sequencing. Using Vsearch (v2.13.4 linux x86 64), the high‐quality sequences were clustered at 97% sequence identity. The Silva database was used to taxonomically classify the high‐quality sequences (version 123). The Chao 1 index (Chao, 1984), Good's coverage (Good, 1953), and the Shannon index (Shannon, 1948) were estimated using the diversity plugin. The sequences of the study have been deposited in the NCBI database under accession number PRJNA794034.

### 2.3 Data analysis

Statistical analysis was performed using IBM SPSS Statistics v19 software (IBM Corp.). One‐way analysis of variance was used to compare averages between groups using the general linear model approach. Differences among treatments were considered to be significant at *p* < .05 and extremely significant at *p* < .01. The results were expressed as the mean ± standard error of the mean (SEM).

## 3 RESULTS

### 3.1 Growth performance

As shown in Table 3, the body weight (BW) did not differ significantly among treatment groups on day 0. However, the BW of the AMP, TB, and AMP&TB groups were significantly lower than those of the CON group on day 30 (*p* < .05), while the BW did not differ significantly among the AMP, TB, and AMP&TB groups (*p* > .05). At weaning (day 65), the BWs of the AMP and TB groups were significantly greater than those of the CON and AMP&TB groups (*p* < .05). The BW did not differ significantly among treatment groups on days 120 and 180 (*p* > .05) but tended to be greater in the AMP and TB groups than in the CON and AMP&TB groups.

**TABLE 3:** Effect of antimicrobial peptide and tributyrin on growth performance of calves.

Item	Treatments				SEM	*p*‐Value
	CON	AMP	TB	AMP&TB		
BW (kg)						
Day 0	39.88	38.78	40.29	39.13	0.47	.707
Day 30	67.29^c^	61.84^ab^	63.48^b^	60.61^a^	0.65	.05
Day 65	93.74^a^	101.70^b^	101.31^b^	91.65^a^	1.14	.05
Day 90	125.88^b^	122.65^ab^	126.72^b^	114.81^a^	1.77	.05
Day 120	152.42	151.70	151.54	144.15	2.59	.67
Day 180	230.50	233.93	241.50	220.42	4.18	.61
ADFI (g)						
Day 4–30	65.24	48.04	51.32	54.99	4.38	.627
Day 30–65	306.28	322.69	314.57	313.99	15.88	.253
ADG (kg)						
Day 0–30	0.84^b^	0.80^ab^	0.77^ab^	0.68^a^	0.02	.05
Day 30–65	0.90^ab^	0.98^b^	0.97^b^	0.83^a^	0.02	.05
Day 65–90	1.00^ab^	1.07^ab^	1.12^b^	0.93^a^	0.03	.05
Day 90–120	0.95	1.00	1.00	0.95	0.04	.94
Day 120–180	1.41	1.36	1.36	1.37	0.03	.94

*Note*: Different superscript letters (a and b) within a row indicate significant differences between treatment groups (*p* < .05).

Abbreviations: ADFI, average daily feed intake; ADG, average daily gain; AMP, antimicrobial peptide; AMP&TB, antimicrobial peptide and tributyrin; BW, body weight; CON, control; TB, tributyrin.

Table 3 displays the ADFI and the ADG among the four treatment groups during each period. There were no significant differences in the ADFI among the four treatment groups. However, the ADG of the CON group was significantly higher than that of the AMP&TB group from day 0 to day 30 (*p* < .05) and the AMP and TB groups had significantly higher ADG values than the CON and AMP&TB groups from day 31 to day 65 (*p* < .05). The ADG values did not differ significantly among treatment groups from day 65 to day 180 (*p* > .05).

As shown in Table 4, diarrhea incidence did not differ significantly between treatment groups from days 4–30 (*p* > .05) but was significantly lower in the AMP and TB groups than in the CON and AMP&TB groups between days 31 and 65 (*p* < .05).

**TABLE 4:** Effects of antimicrobial peptide and tributyrin on diarrhea incidence in calves.

Days	Treatments				SEM	*p*‐Value
	CON	AMP	TB	AMP&TB		
Day 4–30, %	4.33	5.29	5.49	5.50	0.50	.83
Day 30–65, %	1.43^b^	0.32^a^	0.48^a^	1.07^b^	0.27	<.05

*Note*: Different superscript letters (a and b) within a row indicate significant differences between treatment groups (*p* < .05).

Abbreviations: AMP, antimicrobial peptide; AMP&TB, antimicrobial peptide and tributyrin; CON, control; TB, tributyrin.

### 3.2 Immunity and antioxidant indices in serum

On day 75, calves in the AMP&TB group exhibited significantly higher serum IgA levels (*p* < .01) than calves in the other treatment groups, but serum IgA levels did not differ significantly among treatment groups on days 45 and 60 (*p* > .05) (Table 5). Meanwhile, the serum IgM levels were significantly higher in the TB group than those in the other treatment groups on day 45 (*p* < .01). On day 60, the serum IgM levels were significantly higher in the AMP, TB, and AMP&TB groups than those in the CON group (*p* < .01) but did not differ significantly among treatment groups on day 75 (*p* > .05) (Table 5). Serum IgG levels did not differ significantly among treatment groups during each time period (*p* > .05) (Table 5).

**TABLE 5:** Effects of antimicrobial peptide and tributyrin on the immunoglobulin production of calves.

Item	Treatments				SEM	*p*‐Value
	CON	AMP	TB	AMP&TB		
IgA (μg mL^−1^)						
Day 45	2954.72	2964.44	2947.78	3055.00	21.28	.27
Day 60	2947.24	2850.48	2889.76	2911.19	27.20	.57
Day 75	2713.57^a^	2733.47^a^	2771.90^a^	3227.14^b^	56.10	<.01
IgM (μg mL^−1^)						
Day 45	1733.15^a^	1706.00^a^	1987.78^b^	1718.33^a^	30.60	<.01
Day 60	1781.36^a^	1975.91^b^	2127.95^c^	2085.00^c^	34.87	<.01
Day 75	1753.00	1719.50	1765.75	1770.57	12.37	.54
IgG (mg mL^−1^)						
Day 45	8.35	7.96	8.11	8.11	0.31	.09
Day 60	7.28	7.09	7.35	7.18	0.09	.75
Day 75	7.52	7.34	7.34	7.39	0.09	.92

*Note*: Different superscript letters (a, b, and c) within a row indicate extremely significant differences between treatment groups (*p* < .01).

Abbreviations: AMP, antimicrobial peptide; AMP&TB, antimicrobial peptide and tributyrin; CON, control; IgA, immunoglobulin A; IgG, immunoglobulin G; IgM, immunoglobulin M, TB, tributyrin.

As shown in Table 6, on day 45, the serum ROS levels were significantly higher in the AMP and AMP&TB groups than those in the CON group. On day 60, the serum ROS levels were lower in the AMP&TB group than those in the other treatment groups. On day 75, the serum ROS levels were significantly lower in the TB and AMP&TB groups than in the CON group (*p* < .01).

**TABLE 6:** Effects of antimicrobial peptide and tributyrin on the oxidative stress of calves.

Item	Treatments				SEM	*p*‐Value
	CON	AMP	TB	AMP&TB		
ROS (IU mL^−1^)						
Day 45	515.88^a^	554.69^b^	506.58^a^	590.94^c^	9.24	<.01
Day 60	545.84^b^	519.17^b^	528.64^b^	419.92^a^	10.54	<.01
Day 75	576.98^c^	547.67^bc^	527.41^b^	391.59^a^	15.36	<.01
SOD (ng mL^−1^)						
Day 45	7.67^bc^	7.34^ab^	7.15^a^	7.87^c^	0.08	<.01
Day 60	7.45^a^	7.62^ab^	7.86^b^	7.91^b^	0.06	<.01
Day 75	7.43^a^	8.79^b^	8.83^b^	9.10^b^	0.13	<.01
GSH‐Px (ng mL^−1^)						
Day 45	3389.29	3462.50	3557.50	3397.92	40.60	.53
Day 60	3618.75	3512.50	3443.75	3593.75	31.07	.19
Day 75	4155.00^ac^	4355.89^ab^	4173.75^c^	4426.88^b^	35.85	<.01
MDA (nmol mL^−1^)						
Day 45	9.28	9.25	9.23	9.25	0.05	.99
Day 60	9.45^b^	8.86^a^	9.61^b^	10.35^c^	0.13	<.01
Day 75	9.35^b^	9.17^b^	8.57^a^	9.37^b^	0.09	<.01

*Note*: Different superscript letters (a, b, and c) within a row indicate extremely significant differences between treatment groups (*p* < .01).

Abbreviations: AMP, antimicrobial peptide; AMP&TB, antimicrobial peptide and tributyrin; CON, control; GSH‐Px, glutathione peroxidase; MDA, malondialdehyde; ROS, reactive oxygen species; SOD, superoxide dismutase; TB, tributyrin.

Additionally, on day 45, the serum SOD levels were significantly lower in the TB group than in the CON group (*p* < .01) (Table 6). On day 60, the serum SOD levels were significantly higher in the TB and AMP&TB groups than those in the CON group. On day 75, serum SOD levels increased significantly in the AMP, TB, and AMP&TB groups compared to those in the CON group (*p* < .01).

Moreover, on day 75, the serum GSH‐Px levels were significantly lower in the TB group but higher in the AMP&TB group than those in the CON group (*p* < .01) (Table 6). However, serum GSH‐Px levels did not differ significantly among treatment groups on days 45 and 60 (*p* > .05) (Table 6).

Finally, on day 45, the serum MDA levels did not differ significantly between treatment groups (*p* > .05) (Table 6). On day 60, the MDA levels decreased significantly in the AMP group compared to those in the other treatment groups, but the MDA levels increased significantly in the AMP&TB group (*p* < .01) compared to those in the CON group. On day 75, the MDA levels decreased significantly in the TB group compared to those in the other treatment groups (*p* < .01).

### 3.3 Intestinal microflora

Table 7 displays the effect of AMP and TB supplementation on the alpha diversity of the fecal microflora of calves. Alpha diversity analysis, which is often used to characterize species richness and diversity, is represented by indicators such as the Chao 1 index (species richness), the Shannon index (species diversity), and the Coverage index (species coverage). Higher Chao 1 and Shannon index values reflect a richer and more diverse bacterial community in the calf gut. As shown in Table 7, values of the Chao 1, Shannon, and Coverage indices did not differ significantly among treatment groups, indicating that AMP and TB addition had no effect on the diversity and richness of the intestinal microflora.

**TABLE 7:** Effects of antimicrobial peptide and tributyrin on alpha diversity of calf fecal microflora.

Item	Treatments				SEM	*p*‐Value
	CON	AMP	TB	AMP&TB		
Chao 1	4843.57	4170.96	4287.37	5183.02	601.08	.08
Shannon	9.54	9.01	9.00	9.06	0.36	.17
Coverage	0.97	0.98	0.98	0.97	0.00	.11

Abbreviations: AMP, antimicrobial peptide; AMP&TB, antimicrobial peptide and tributyrin; CON, control; TB, tributyrin.

Table 8 presents the genus‐level composition of the fecal bacterial communities of the four treatment groups. Compared with the CON group, in the AMP group, the relative abundance of *Rikenellaceae_RC9_gut_group*, *Ruminococcaceae_UCG‐014*, *[Eubacterium]_coprostanoligenes_group*, and *Parabacteroides* was significantly increased (*p* < .01), whereas that of *Ruminococcaceae_UCG‐005*, *Faecalibacterium*, and *Christensenellaceae_R‐7_group* was significantly decreased (*p* < .01). Compared with the CON group, the TB group displayed a significant increase in the relative abundance of *Rikenellaceae_RC9_gut_group*, *Ruminococcaceae_UCG‐005*, and *Bacteroides* abundance (*p* < .01), and a significant decrease (*p* < .01) in the relative abundance of *Ruminococcaceae_UCG‐014*, *Faecalibacterium*, *[Eubacterium]_coprostanoligenes_group*, *Blautia*, and *Christensenellaceae_R‐7_group*. Meanwhile, the relative abundance of *Rikenellaceae_RC9_gut_group* and *Bacteroides* was significantly increased in the AMP&TB group compared with that in the AMP and TB groups (*p* < .01). In contrast, the relative abundance of *Ruminococcaceae_UCG‐014*, *Faecalibacterium*, *[Eubacterium]_coprostanoligenes_group*, and *Christensenellaceae_R‐7_group* was significantly reduced in the AMP&TB group compared with that in the AMP and TB groups (*p* < .01).

**TABLE 8:** Effects of antimicrobial peptide and tributyrin on calf fecal microflora on the genus level.

Genus	Treatments				*p*‐Value
	CON	AMP	TB	AMP&TB	
*Ruminococcaceae_UCG‐005*	13.94^b^	9.70^a^	20.52^c^	14.30^b^	<.01
*Rikenellaceae_RC9_gut_group*	3.77^a^	9.85^b^	14.13^c^	24.57^d^	<.01
*Bacteroides*	9.45^a^	11.15^ab^	13.73^b^	17.21^c^	<.01
*Ruminococcaceae_UCG‐014*	13.71^a^	15.57^b^	9.44^c^	4.09^d^	<.01
*Faecalibacterium*	7.04^d^	4.79^b^	6.03^c^	3.26^a^	<.01
*Muribaculaceae*	5.51^b^	3.67^a^	3.15^a^	7.20^c^	<.01
*[Eubacterium]_coprostanoligenes_group*	4.05^b^	4.99^c^	3.74^b^	2.57^a^	<.01
*Christensenellaceae_R‐7_group*	6.24^d^	3.65^c^	3.04^b^	2.17^a^	<.01
*Parabacteroides*	1.87^a^	2.53^b^	1.51^a^	2.47^b^	<.01
*Blautia*	2.72^b^	2.64^b^	1.11^a^	0.99^a^	<.01

*Note*: Different superscript letters (a, b, and c) within a row indicate extremely significant differences between treatment groups (*p* < .01).

Abbreviations: AMP, antimicrobial peptide; AMP&TB, antimicrobial peptide and tributyrin; CON, control; TB, tributyrin.

## 4 DISCUSSION

Recent studies have reported that as substitutes for antibiotics, AMP and TB can improve animal growth performance (Wang et al., 2019) and immune function (Jang et al., 2017) and regulate the balance of gastrointestinal microbes (Yang et al., 2018). However, most studies have focused on piglets and broilers and only a few studies have explored the effects of AMP and TB on ruminants. Nevertheless, some studies have shown that AMP and TB have positive effects on ruminants, reporting that AMP and TB supplementation significantly increased ADG values and decreased fecal scores (Leeson et al., 2005; Liu et al., 2017). The present study demonstrated that AMP and TB supplementation significantly increased ADG values and BW, while decreasing average fecal scores in weaned calves, in addition to promoting their subsequent growth performance. These results are consistent with the findings of previous studies in which dietary AMP and TB increased growth performance and decreased diarrhea incidence in animals (Hu et al., 2017). The ineffective ratio of AMP to TB may be the reason why supplementation with AMP and TB did not improve the growth performance of calves in the present study.

The intestinal microflora composition plays an important role in gut health (Hooper et al., 2012). However, limited data have been reported to date regarding the effects of AMP and TB on the intestinal microflora of animals. In this study, the top 10 genera of bacteria detected in abundance were compared among different groups, among *Ruminococcaceae_UCG‐005*, *Ruminococcaceae_UCG‐014*, *Blautia* and *[Eubacterium]_coprostanoligenes_group* are Gram‐positive bacterium. *Rikenellaceae_RC9_gut_group*, *Bacteroides*, *Faecalibacterium*, *Muribaculaceae*, *Christensenellaceae_R‐7_group*, and *Parabacteroides* are Gram‐negative bacterium. *Ruminococcaceae_UCG‐005*, *Ruminococcaceae_UCG‐014*, *Faecalibacterium*, *[Eubacterium]_coprostanoligenes_group*, and *Parabacteroides* are fiber‐degrading bacteria that can ferment fiber and produce short‐chain fatty acids (SCFAs). SCFAs have multiple beneficial effects on animal performance and intestinal health (Guilloteau et al., 2010). Previous studies have indicated that SCFAs help maintain the mechanical barrier provided by the intestinal epithelium by upregulating the expression of tight junction proteins such as zonula occludens 1, claudin‐3 and ‐4, and occludin (Bhat et al., 2019; Gao et al., 2017; Jirsova et al., 2019). SCFAs can also inhibit inflammatory responses by downregulating pro‐inflammatory factors, such as TNF‐α, IL‐17A, and IL‐6 (Guilloteau et al., 2010), while upregulating anti‐inflammatory factors, such as IL‐18 (Kalina et al., 2002). In addition, some members of the gut microbiota exert direct anti‐inflammatory effects. For example, *Ruminococcaceae_UCG‐014*, *[Eubacterium]_coprostanoligenes‐group*, and *Christensenellaceae_R‐7_group* can increase the activity of nitric oxide (NO) synthase by regulating the MyD88 pathway, thereby upregulating NO levels and inhibiting inflammatory responses (Kubinak et al., 2015). Moorthy et al. studied the use of polyphenols as a prebiotic treatment for high‐fat diet‐induced obesity, demonstrating that *Blautia* exerts anti‐inflammatory effects (Moorthy et al., 2021). *Rikenellaceae_RC9_gut_group*, the dominant bacteria in the ruminant gut, can degrade mucin. A previous study reported that *Rikenellaceae_RC9_gut_group* was significantly and negatively correlated with the inflammation‐related indicator IFN‐γ (Haberman et al., 2019). In the present study, supplementation with AMP significantly increased the relative abundance of *Rikenellaceae_RC9_gut_group*, *Ruminococcaceae_UCG‐014*, and *[Eubacterium]_coprostanoligenes‐group*, while supplementation with TB significantly increased the relative abundance of *Rikenellaceae_RC9_gut_group*, *Ruminococcaceae_UCG‐005*, and *Bacteroides*. However, the combined use of AMP and TG increased the relative abundance of *Rikenellaceae_RC9_gut_group* and *Bacteroides* but decreased the relative abundance of most beneficial bacteria. Thus, the study findings suggest that the enhanced growth performance and lower diarrhea incidence among calves supplemented with AMP or TG may be attributed to their improved intestinal microflora.

Changes in the structure and abundance of the gut microbial community influence not only the host's disease status, nutrient absorption, and metabolism but also the host's immune function (Kim & Isaacson, 2015; Pajarillo et al., 2015; Senghor et al., 2018; Sonnenburg & Bäckhed, 2016). Immunoglobulin A (IgA) mainly exists in mucosal defense systems, such as respiration, digestion, and reproduction is relatively low in serum. Its concentration is relatively low in serum. Therefore, it plays an important role in frontline anti‐infection defense. Immunoglobulin M (IgM) with the highest molecular weight is the earliest immunoglobulin produced after infection or immunity in animals, belonging to the initial immune response. Immunoglobulin G (IgG) is the main immunoglobulin produced in the humoral immune response, with the highest concentration in serum, accounting for 75%–80% of the total immunoglobulin content. It plays a role in anti‐infection, neutralizing toxins, and regulating (Ulfman et al., 2018). IgA, IgG, and IgM are produced by B lymphocytes and participate in humoral immunity. Their concentrations can be used as a key parameter to reflect an animal's immune status (Bedford & Gong, 2018; Ulfman et al., 2018). Prgomet et al. reported that adding lactoferrin, an AMP derived from milk protein, to calf diets resulted in increased IgG levels (Prgomet et al., 2007). This result seems inconsistent with the findings of the present study, in which serum IgA and IgG levels were not significantly affected by AMP supplementation. However, a previous study reported that the addition of dietary AMP had no effect on IgA and IgG contents in calf serum but only increased IgM levels (Shan et al., 2007). The reason for the discrepancy in these results may be the different types of AMP and animal species used in the studies. Moreover, a previous study with mice reported that dietary TB increased serum IgM levels (Leonel et al., 2013), which concurs with the results of the present study. IgM is the earliest immune response produced by the body and is essential for maintaining the health of the body (Ehrenstein & Notley, 2010). The normal range of IgM is .48–2.12 μg m^−1^. An increase in IgM in the normal range indicates improved immune capacity of the body (Fomenky et al., 2017). Similar results were also obtained in this experiment: the IgM level in blood was significantly increased compared with that in the control, but the increase was basically within the normal range. These reports support that AMP and TB likely have a positive effect on the secretion of immunoglobulins in animals.

A dynamic equilibrium between oxidation and anti‐oxidation is maintained under normal circumstances, but this balanced state can be broken when the animal is subjected to external stimuli, thereby inducing oxidative stress. ROS are oxygen‐containing, highly chemically reactive chemicals that play important roles in cell signaling and homeostasis. SOD and GSH‐Px are important antioxidant enzymes that maintain redox balance in animals, which could eliminate ROS and reduce the formation of lipid peroxides. MDA is the final product of lipid oxidation in the body and can reflect the degree of lipid peroxidation and indirectly reflect the extent of cell damage (Lepage et al., 1991; Viarengo et al., 1995). MDA has toxic effects when cross‐linked with lipoproteins. In the current study, the addition of AMP and TB ameliorated the damage caused by oxidative stress in calves, which was manifested by a significant decrease in ROS and MDA levels, as well as increased SOD and GSH‐Px activities. Similarly, a previous study reported that supplementation with AMP enhanced the activities of SOD and GSH‐Px, as well as total antioxidant capacity (T‐AOC), in piglets (Tang et al., 2009). Moreover, a study reported that dietary TB promoted intestinal mucus production and improved intestinal oxidative stress, suggesting that TB can effectively increase T‐AOC, GSH‐Px, and SOD activities and reduce ROS and MDA levels (Wang et al., 2019). Interestingly, combined supplementation with AMP and TB resulted in reduced ROS levels and increased SOD levels, but MDA levels remained high. These results indicate that the combined use of AMP and TB does not improve the antioxidant capacity of calves and will not alleviate the damage caused by oxidative stress over time. These conflicting results may be due to poor matching between AMP and TB. To obtain the desired result, more investigation is required into the optimal ratios of AMP and TB.

## 5 CONCLUSION

In conclusion, compared with dietary supplementation with essential oils, dietary supplementation with AMP and TB could increase the relative abundance of intestinal microorganisms and inhibit the growth of intestinal flora associated with pro‐inflammatory responses, helping to improve the intestinal barrier function. Dietary supplementation with AMP and TB could also enhance the immune function of calves and their ability to resist oxidative stress, ultimately promoting the growth of calves. However, compared to supplementation with either AMP or TB, supplementation with AMP and TB together inhibited the growth of beneficial gut microflora, while increasing the relative abundance of unfavorable bacteria, resulting in aggravated damage and poorer growth performance.

## AUTHOR CONTRIBUTIONS

**Junling Gao:** Data curation (equal); formal analysis (equal); investigation (equal); software (equal); writing – original draft (equal). **Jianan Dong:** Data curation (equal); formal analysis (equal); investigation (equal). **Zhe Sun:** Methodology (equal). **Tao Wang:** Validation (equal); writing – review and editing (equal). **Yanling Guan:** Resources (equal). **Yue Sun:** Resources (equal). **Guixin Qin:** Writing – review and editing (equal). **Yuguo Zhen:** Conceptualization (equal); project administration (equal); validation (equal). **xuefeng zhang:** Conceptualization (equal); supervision (equal); validation (equal); writing – review and editing (equal).

## FUNDING INFORMATION

This study was supported by the Jilin Province Feed Engineering and Technology Research Center (20170623075TC).

## CONFLICT OF INTEREST STATEMENT

The authors declare no conflict of interest.

## ETHICAL APPROVAL

The animal study protocol was performed according to Guidelines for the Care and Use of Experimental Animals of Jilin Agricultural University (JLAU‐ACUC2017‐006).

## Data Availability

The data that support the findings of this study are available from the corresponding author upon reasonable request.
